# Mutation spectrum and genotype-phenotype correlations in a large French cohort of MYH9-Related Disorders

**DOI:** 10.1002/mgg3.68

**Published:** 2014-02-07

**Authors:** Béatrice Saposnik, Sylvie Binard, Odile Fenneteau, Alan Nurden, Paquita Nurden, Marie-Françoise Hurtaud-Roux, Nicole Schlegel, on behalf of the French MYH9 network

**Affiliations:** 1 Service d'Hématologie Biologique and National Reference Center on Inherited Platelet Disorders, Hôpital Robert-Debré48 Boulevard Sérurier, 75019, Paris, France; 2 Institut Hospitalo-Universitaire LIRYC, Hôpital Xavier ArnozanPessac, France

**Keywords:** Inherited platelet disorders, leukocyte inclusions, macrothrombocytopenia, mutation analysis, MYH9-RD

## Abstract

MYH9-Related Disorders are a group of rare autosomal dominant platelet disorders presenting as nonsyndromic forms characterized by macrothrombocytopenia with giant platelets and leukocyte inclusion bodies or as syndromic forms combining these hematological features with deafness and/or nephropathy and/or cataracts. They are caused by mutations in the *MYH9* gene encoding the nonmuscle myosin heavy chain II-A (NMMHC-IIA). Until now, at least 49 *MYH9* mutations have been reported in isolated cases or small series but only rarely in large series. We report the results of an 8-year study of a large cohort of 109 patients from 37 sporadic cases and 39 unrelated families. We have identified 43 genetic variants, 21 of which are novel to our patients. A majority, 33 (76.7%), were missense mutations and six exons were preferentially targeted, as previously published. The other alterations were three deletions of one nucleotide, one larger deletion of 21 nucleotides, and one duplication. For the first time, a substitution T>A was found in the donor splice site of intron 40 (c.5765+2T>A). Seven patients, four from the same family, had two genetic variants. The analysis of the genotype-phenotype relationships enabled us to improve the knowledge of this heterogeneous but important rare disease.

## Introduction

It is now well established that a group of autosomal dominant inherited thrombocytopenias with large/giant platelets and leukocyte inclusions, initially designed as May Hegglin Anomaly (MHA: MIM 155100), Sebastian, Fechtner or Epstein Syndrome (SBS: MIM 605249, FTNS: MIM 153640, or EPTS: MIM 153650, respectively) are in fact a clinical continuum associated with mutations in the *MYH9* gene. Now called MYH9-Related Disorders (MYH9-RD: MIM 160775), they are thus characterized by significant phenotypic and genotypic heterogeneity (Heath et al. 2001; Balduini et al. 2002; Seri et al. 2003). MHA and SBS were previously called “nonsyndromic” because the anomalies are restricted to blood cells, platelets, and leukocytes. The other disorders, FTNS, EPTS, and APSMT, were said to be “syndromic” because the hematological symptoms, macrothrombocytopenia and leukocyte inclusions, also called Döhle-like bodies, were associated with various combinations of extrahematological symptoms: sensorineural hearing loss and nephritis in EPTS with, in addition, cataracts in FTNS. MYH9 mutations have also been found in two families with another inherited disorder, DFNA 17 syndrome, characterized by hearing loss without thrombocytopenia (Lalwani et al. 2000; Hildebrand et al. 2006).

*MYH9* is a large gene composed of 41 exons and is located on chromosome 22 q12-13. It encodes the nonmuscle myosin heavy chain IIA (NMMHC-IIA, MYH9) that is the only myosin present in platelets. NMMHC-IIA is a protein of 230 kDa. As a motor protein, it plays a crucial role in the contractile and secretory functions of platelets (Sellers 2000). The first exon is not translated. Exons 2–19 of *MYH9* encode the NMMHC-IIA N-terminal or motor domain (MD), an actin-binding site and the ATP hydrolysis region. Exon 20 encodes the “neck” that transfers the force generated by the MD and binds the myosin light chains. Exons 21–40 encode the coiled-coil terminal or tail domain (TD), crucial for heavy chain dimerization and the assembly of myosin filaments. Exon 41 encodes the nonhelical C-terminal sequence, a phosphorylation and regulatory domain.

Since the discovery of the association of MYH9 with these disorders (Seri et al. 2000; Heath et al. 2001; Kunishima et al. 2001), several series of patients have been reported (Dong et al. 2005; Pecci et al. 2008; Althaus and Greinacher 2009) as well as a number of isolated cases, in populations from different continents. A recent review reported at least 44 different *MYH9* mutations in 218 MYH9-RD unrelated families (Balduini et al. 2011). This was later updated to 49 mutations (Balduini et al. 2013). Among the 14 exons in which mutations have been identified, six (exons 2, 17, 27, 31, 39, 41) are affected preferentially. The majority of mutations are missense mutations and all mutations predicting a truncated NMMHC-IIA are located in the last exon. Small deletions or duplications are rare and only one large deletion has been identified (Kunishima et al. 2008). Significantly, 20–35% of the mutations appear to be de novo. Only two cases have been shown to be due to somatic or germinal mosaicism (Kunishima et al. 2005, 2009). Analysis of one of the largest series of MYH9-RD patients led Pecci et al. (2008) to conclude that the location of the mutation on *MYH9* might predict the severity of the clinical evolution. It was claimed that mutations predicting substitutions in the MD of the protein are more frequently associated with extrahematological symptoms than those predicting substitutions in the TD. Since 2008, this hypothesis is generally accepted. However, a larger number of patients studied over a long period are needed to confirm this prediction (Saito and Kunishima 2009). In addition, for those cases that do not follow this prediction because they present with a MD mutation but no extrahematological symptoms or in contrast with TD mutation but with deafness, and/or nephropathy and/or cataracts, it was suggested that other factors might interact with NMMHC-IIA functions (Althaus and Greinacher 2009).

We now report the results of an 8-year study of MYH9-RD in France leading to the identification of a series of 109 patients from 37 sporadic cases and 39 unrelated families from among 295 patients with inherited macrothrombocytopenia analyzed for mutations in MYH9 in our center after phenotypic exclusion of Bernard-Soulier syndrome, Gray Platelet syndrome, Platelet-Von Willebrand Disease and Paris-Trousseau syndrome. We report 43 genetic variants and have analyzed genotype-phenotype relationships that give rise to a more complete picture of this highly heterogeneous but important rare disease.

## Subjects, Materials, and Methods

### Cohort of MYH9-RD patients and family members

A cohort of patients with a phenotype compatible with MYH9-RD was enrolled in the study during 8 years. Inclusion criteria were: chronic macrothrombocytopenia suspected to be inherited because of the absence of a known acquired cause, the presence or absence of a family history of thrombocytopenia and the phenotypic exclusion of other known inherited macrothrombocytopenia. When the presence of a *MYH9* mutation was genetically confirmed in a propositus, symptomatic or asymptomatic family members who accepted to participate to the study were also enrolled. The propositi and their relatives were recruited on a national basis within different sites in France.

The available clinical history of the patients was recorded including age and nature of initial symptom(s), platelet count (extent of thrombocytopenia), platelet size, and morphology, presence or absence of leukocyte inclusions, bleeding episodes, presence and age at onset of nephritis, deafness or cataracts. Bleeders were defined as suffering from spontaneous muco-cutaneous manifestations including easy bruising, epistaxis, gum bleeding, hematomas, ecchymosis, petechial purpura, menometrorrhagias, or provoked bleeding during/after trauma, tooth extraction, minor or major surgery, pregnancy, delivery and postpartum. Nonbleeders were those patients who did not present any bleeding episodes, spontaneous or provoked. Nephritis included several stages of kidney damage. Proteinuria meant a level of proteinuria over 0.5 g/24 h. Chronic renal failure (CRF)-associated proteinuria and increased serum creatinine level and/or decreased estimated glomerular renal filtration (GRF) according to age. End-stage renal failure (ESRF) corresponded to chronic dialysis that could lead to kidney transplantation in some patients. Deafness was defined by an auditive examination and could lead to hearing help in some cases. In young children, hearing loss was defined using sensory evoked potentials. Cataract diagnosis was established as the presence of lens opaque spots at ophthalmological examination. The time between the initial symptoms and the genetic diagnosis of MYH9-RD was also noted and clinical data from family members were collected when possible.

All the participants gave their written informed consent and the study was approved by the Ethical Committee of the Robert-Debré University Hospital Center, Paris.

### Platelet count and size

The platelet count of all patients and family members was measured by the hematology analyzer routinely used in each center. In early studies using impedance analyzers, the platelet count was controlled by light microscopy “(microscope count)”. More recently, most results were obtained using optical light scatter analyzers “(optical count)” alone. Platelet morphology was studied on May-Grunwald-Giemsa (MGG)-stained blood smears. When possible, platelet size was reevaluated at the Robert-Debré Centre and compared to that of erythrocytes using optical microscopy. This allowed platelets to be classified into three groups: the first group represented platelets of normal size, the second group consisted of giant platelets defined by a size equal to or above that of erythrocytes, and the third group consisted of intermediate-sized platelets whose size was intermediate between the normal and the giant ones. Results for each patient were expressed as a percentage of each group.

### Leukocyte myosin inclusions

The MGG-stained blood smears were carefully examined for the presence or absence of basophilic inclusions (Döhle-like bodies) in the leukocyte cytoplasm. Whenever possible, the percentage of neutrophils with intracytoplasmic inclusions was recorded as well as the shape, number, size, staining intensity, and location of the inclusions.

The presence or absence of NMMHC-IIA aggregates was studied using an immunofluorescence (IF) technique adapted from Kunishima et al. (2003). Briefly, smears from ethylenediaminetetraacetic acid (EDTA)-anticoagulated blood were carefully air-dried and directly treated or fixed with acetone before being stored at −20°C. The leukocyte membranes were permeabilized with a mixed acetone/methanol solution (vol:1/1) at −20°C during 3 min. Thawing was performed at 4°C and room temperature successively. Slides were incubated with goat serum, then anti-human nonmuscle myosin-IIA rabbit polyclonal antibody (BT561 and BT567, Biomedical Technology, CliniSciences, Stoughton, MA) at the selected dilution, and finally with fluorochrome-labeled goat anti-rabbit antibody (Goat Anti-Rabbit IgG-TRIT, CliniSciences, Stoughton, MA). The cells were then examined under a fluorescence microscope (Olympus BX60, Lille, France). Negative and positive controls were included in each series. Negative controls were selected among healthy individuals. Images were acquired using Genikon software.

### *MYH9* variants screening on genomic DNA

Blood was collected from all patients by venipuncture in tubes containing 1.6 mL EDTA/mL blood. Genomic DNA was extracted from white blood cells using the Qiamp DNA Blood Maxi kit according to the manufacturer's instructions (Qiagen, Courtaboeuf, France).

*MYH9* cDNA according to the GenBank Refseq NM_0024473.4 in NCBI was used as the reference sequence. The entire sequence of the 40 coding exons and exon–intron boundaries of *MYH9* was amplified by polymerase chain reaction (PCR). Primers were designed by us or selected from those already published (Kunishima et al. 2001). Their composition is available on request. PCR amplification of each of the 40 fragments of the *MYH9* gene was performed in a total volume of 50 *μ*L with 50 ng of genomic DNA, 0.4 *μ*mol/L of each primers (Eurogentec, Angers, France), 200 *μ*mol/L of dNTPs (Amersham Biosciences Europe, Orsay, France), 1.25 U ampli Taq (Promega, Charbonnières, France), adapted MgCl_2_ concentrations and 1 mol/L betaine in enzyme-related PCR buffer. Each amplification product was purified by filtration on Millipore plates (Saint Quentin en Yvelines, France) and sequenced with a DNA sequencing kit according to the manufacturer's instructions. Sequence products were purified with Manu N45 multiscreen sequence reaction filter plates (Merck Millipore, Saint Quentin en Yvelines, France) and then run on a 3130 ABI automated sequencer (Applied Biosystems by Life technologies, Foster City, CA). Data were analyzed with Sequencing Analysis (v3.7) software.

Mutations were numbered according to the HGVS recommendations for mutation nomenclature (http//:www.hgvs.org/mutnomen/), exon 1 being as usually published with the A of the ATG translate initiation start site as nucleotide +1. Genetic variants were classified according to the location of the mutation either in the MD (patients [P]: “MDP”) or in the TD (P: “TDP”).

### Statistical analysis

Continuous variables are described as mean and standard deviation (SD) or as median and range. Categorical variables are reported with counts and percentages. Percentages of giant, intermediate-sized, or normal platelets were compared in four groups of patients defined by their platelet count (×10^9^/L): <10, 11–50, 51–99, and 100–136 using the analysis of variance (ANOVA) test (Kruskal-Wallis test). Student unpaired *t-*test for continuous variables was used to compare the platelet count and size between bleeders and nonbleeders and to compare the platelet count between MDP and TDP. Comparisons of the platelet size between bleeders versus nonbleeders according to a platelet count above or under 50 × 10^9^/L, comparisons of the age at onset between deafness and nephropathy, and comparisons of the age at onset of nephropathy or deafness between MDP and TDP were based on the Mann–Whitney test for continuous variables. Comparisons of platelet count between bleeders and nonbleeders at the time of the discovery of macrothrombocytopenia were based on the Mann–Whitney test for continuous variables. The comparison of the platelet count in bleeders versus nonbleeders in two groups, one with platelet count under 50 × 10^9^/L and the other above 50 × 10^9^/L, was expressed using the Mann–Whitney test for continuous variables. The chi-square test or Fisher exact test for categorical variables was used to compare the percentage of bleeders with a platelet count above or under 50 × 10^9^/L and to compare the results between MDP and TDP. The chi-square test was also used to compare the total number of patients with extrahematological symptoms or the number of patients with nephropathy or deafness according to the location of the mutation in the MD or TD. Statistical tests were run on graphpad prism 5.0 (Logilabo, Paris, France) and differences with *P* values less than 0.05 were considered statistically significant.

## Results

### Population characteristics

This study includes 76 propositi with a genotype confirming MYH9-RD and 85 family members from 39 unrelated families. Among the 85 family members, 33 had a MYH9 mutation. The demographic characteristics of the cohort are presented in Table 1. This led to the enrollment of a total of 109 patients of which 74 were adults (54 propositi and 20 family members) and 35 were children (22 propositi and 13 family members) with seven under 1 year of age. The age at which macrothrombocytopenia was first reported was available for 74 patients (Table S1, panel A) and included 52 children and 22 adults, indicating that in our cohort, macrothrombocytopenia was discovered during childhood for a majority of patients (70.3%) half of whom were under 5 years of age (52.7%). Macrothrombocytopenia was discovered in three situations (Table S1, panel B): following bleeding (29.7%), in known familial cases of thrombocytopenia/macrothrombocytopenia (24.4%) and by systematic platelet counting before surgery or during infection (45.9%). No patient presented extrahematological manifestations as initial symptoms except one who was discovered with congenital cataracts of unknown origin. A total of 29 patients (41.4% from 70 with available data) were initially misdiagnosed as having Immune Thrombocytopenic purpura (ITP). For these patients, corticosteroids (25 cases) or intravenous immunoglobulins (IVIgs) (19 cases) failed to significantly increase platelet count. Among the patients suspected to have ITP, 10 underwent splenectomy, the youngest at 22 months (range: 22 months – 32 years among the six cases for whom the age was available).

**Table 1:** Characteristics of the cohort

	Patients MYH9-RD	Propositi	Affected family members	Non affected family members	Total population*
Number	109	76 Sporadic: 37 Family index: 39	33 (22 unrelated families)	52	161
Women/men	64/45	45/31	19/14	26/26	90/71
Age (years) at genetic diagnosis					
≤1	7	4	3	3	10
1–18	28	18	10	10	38
Adult	74	54	20	39	113

* Origin – Caucasian: 100/109 (91.74%); Kanak: 2 (1.84%); Maghrebin: 6 (5.50%); Malagasy: 1 (0.92%).

### *MYH9* genotype

**Figure 1:**
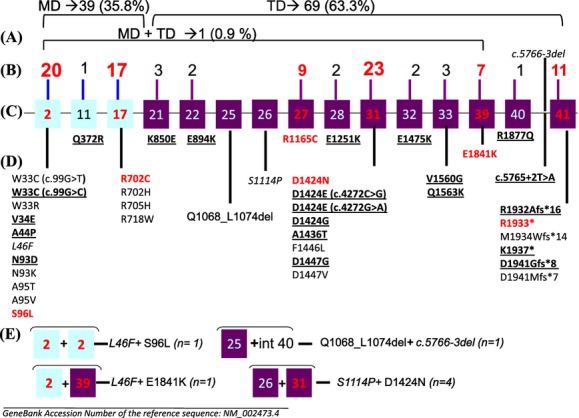
*MYH9* genetic variants in 109 patients. (A) Number and percentage of genetic variants in the two different domains: MD (motor domain) and TD (tail domain). (B) Number of patients with mutations in each exon. In red: the highest number of patients. (C) Affected exons and intron – light blue boxes: exons coding for the MD; purple boxes: exons coding for the TD; numbers in red: exons more frequently affected. (D) Description of mutations per exon – in black, bold, and underlined: novel variants identified only in our patients; in red: more frequently affected variants as reported in the literature and as found in our patients; in italics: SNPs. (E) Double genetic variants.

Among the 109 patients, 39 (35.8%) had a genetic alteration in the MD, 69 (63.3%) in the TD, and one patient (0.9%) had a genetic variant in each domain (Fig. 1A). The distribution of the mutations within affected exons is shown in Figure 1B. Six exons were preferentially affected, 2 in the MD (exon 2 and 17) and 4 in the TD (exons 27, 31, 39, and 41. Their frequency is shown in Figure 1B and C.

The nature of the genetic alterations is summarized in Figure 1D and E. A majority were missense mutations (*n* = 33; 80.5%) spreading over 15 exons and one intron. Two mutations, p.W33C and p.D1424E were each the result of two different nucleotide transitions on the same codon, c.99G>T or c.99G>C in exon 2 and c.4272C>A or c.4272C>G in exon 31, respectively. Two nonsense mutations c.5797C>T and c.5809A>T leading to p.R1933* and p.K1937*, respectively, and one duplication of a G nucleotide (c.5820_5821dup) leading to a frameshift and a premature stop codon p.Asp1941Glyfs*8 were identified in exon 41. Five deletions were identified, one of 21 nucleotides (c.3202_3222del) affecting exon 25 predicted a deletion of seven amino acids in the protein (p.Q1068_L1074del). There were four deletions of one nucleotide, three in exon 41 (c.5794delC, c.5800delA, and c.5821delG) predicting a frameshift and a premature stop codon (p.R1932Afs*16, p.M1934Wfs*14, p.D1941Mfs*7, respectively), and one in intron 40 (c.5766-3del) near the acceptor splice site of exon 41. A substitution T>A was also found in the donor splice site of intron 40 (c.5765+2T>A). Among the 109 patients, seven (6.4%) had two genetic variants (Fig. 1E). Four subjects associating c.3340T>C (p.S1114P) and c.4270G>A (p.D1424N) were from the same family, the father and his three sons. The mutation found in 10 propositi, (c.287C>T [p.S96L]: 3, c.2104C>T [p.R702C]: 1, c.2105G>A [p.R702H]: 1, c.2114G>A [p.R705H]: 1, c.4336T>C [p.F1446L]: 1, c.5521G>A [p.E1841K]: 2, c.5794del [p.R1932Afs*16]: 1 was not identified in the parents suggesting the de novo nature of the mutation. The study of the 11th propositus's family (c.287C>T [p.S96L]) is under progress.

The nucleotide changes in cDNA and corresponding protein alterations are listed in Table 2. Among the 43 genetic variants, a total of 21 (48.8%) were novel to our patients (Table 2).

**Table 2:** MYH9 variants: nucleotide cDNA changes and corresponding protein alterations

Exon	Nucleotide change	Protein alteration	P/FM/F
**2**	c.97T>C	p.W33R	1/1/1
	**c.99G>C**	**p.W33C**	**1/0/0**
	c.99G>T	p.W33C	1/1/1
	**c.101T>A**	**p.V34E**	**1/0/0**
	**c.130G>C**	**p.A44P**	**1/0/0**
	*c.136C>T*	*p.L46F*^1^	3/0/0
	**c.277A>G**	**p.N93D**	**1/0/0**
	c.279C>G	p.N93K	1/1/1
	c.283G>A	p.A95T	2/0/0
	c.284C>T	p.A95V	1/0/0
	c.287C>T	p.S96L	6/1/1
**11**	**c.1115A>G**	**p.Q372R**	**1/0/0**
**17**	c.2104C>T	p.R702C	6/2/1
	c.2105G>A	p.R702H	4/1/1
	c.2114G>A	p.R705H	2/0/0
	c.2152C>T	p.R718W	1/1/1
**21**	**c.2548A>G**	**p.K850E**	**1/2/1**
**22**	**c.2680G>A**	**p.E894K**	**2/0/0**
**25**	c.3202_3222del	p.Q1068_L1074del	1/0/0
**26**	*c.3340T>C*	*p.S1114P*^2^	1/3/1
**27**	c.3493C>T	p.R1165C	2/7/2
**28**	**c.3751G>A**	**p.E1251K**	**1/1/1**
**31**	**c.4272C>G**	**p.D1424E**	**1/2/1**
	**c.4272C>A**	**p.D1424E**	**1/0/0**
	**c.4271A>G**	**p.D1424G**	**2/1/1**
	c.4270G>A	p.D1424N	7/6/4
	**c.4306G>A**	**p.A1436T**	**1/0/0**
	c.4336T>C	p.F1446L	1/0/0
	**c.4340A>G**	**p.D1447G**	**1/0/0**
	c.4340A>T	p.D1447V	2/2/1
**32**	**c.4423G>A**	**p.E1475K**	**2/0/0**
**33**	**c.4679T>G**	**p.V1560G**	**2/0/0**
	**c.4687C>A**	**p.Q1563K**	**1/0/0**
**39**	c.5521G>A	p.E1841K	5/2/2
**40**	**c.5630G>A**	**p.R1877Q**	**1/0/0**
**Intron 40**	**c.5765+2T>A**	p.R1922Rfs^*^43	**1/0/0**
***Intron 40***	*c.5766-3del*		
**41**	**c.5794del**	p.R1932Afs^*^16	**1/0/0**
	c.5797C>T	p.R1933X	4/2/1
	c.5800del	p.M1934Wfs^*^14	1/0/0
	**c.5809A>T**	**p.K1937X**	**1/0/0**
	**c.5820_5821dup**	p.D1941Gfs^*^8	**1/0/0**
	c.5821del	p.D1941Mfs^*^7	1/0/0

GenBank Accession Number of the relevant wild-type MYH9 gene sequence: NM_002473.4. In bold: variants only found in our patients (*n* = 21); In italics: SNP (*n* = 3). Motor Domain, exons 2 to 17: 16 variants; Tail Domain, exons 21 to 41: 27 variants. P, propositi; FM, number of affected family members; F, number of families with a propositus, and affected family members.

1 p.L46F alone: 1 proposita, sporadic case; p.L46F+p.S96L: 1 proposita, sporadic case; p.L46F+p.E1841K: 1 proposita with one daughter with p.E1841K alone.

2 p.S1114P associated with p.D1424N in the propositus and his three sons.

### Phenotype

#### Macrothrombocytopenia

**Figure 2:**
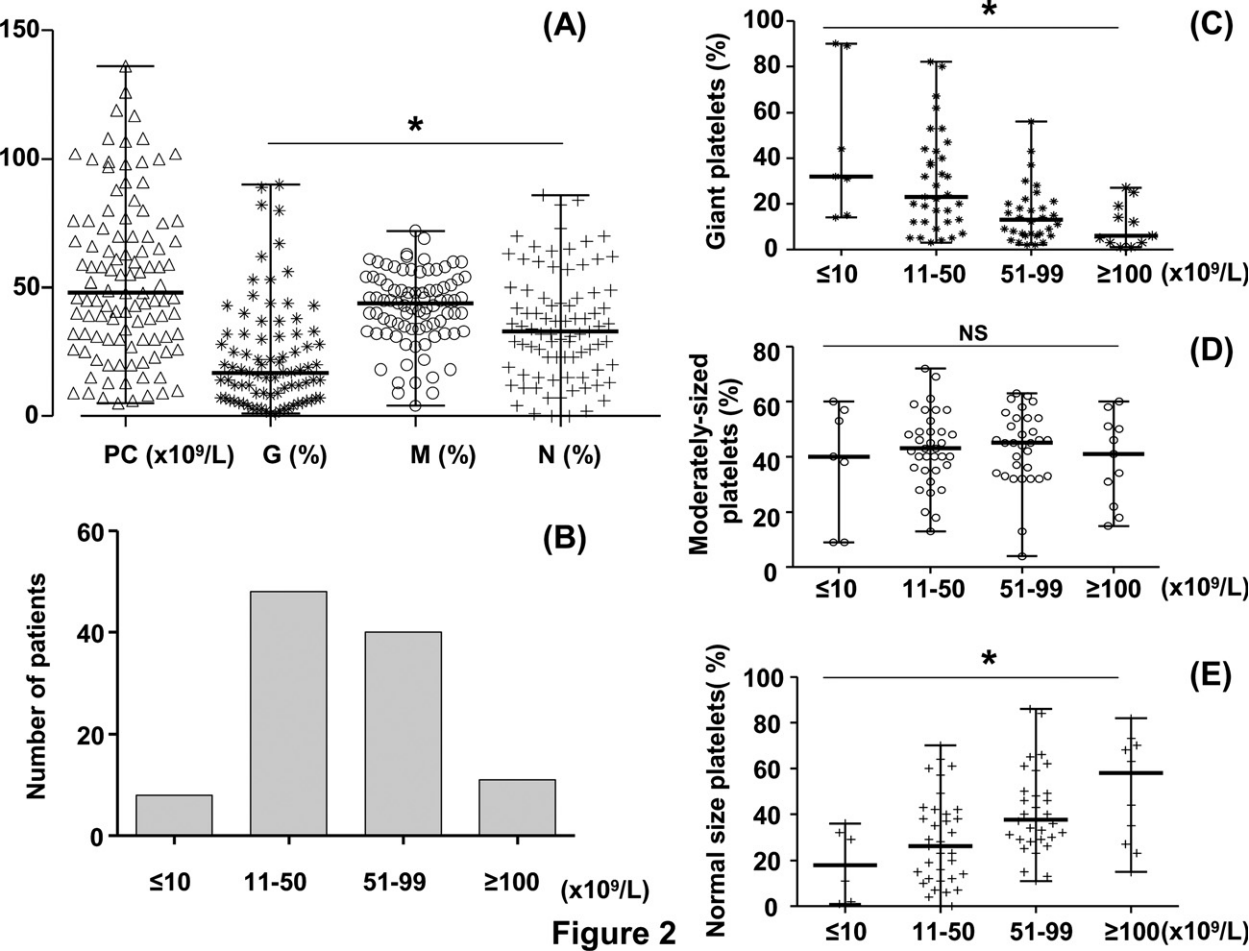
Platelet characteristics in the 109 patients. (A) Platelet count (PC) and size [median and range]. PC – platelet count analyzer (×10^9^/L): [48 and <10–136], (mean ± SD: 53.2 ± 30.7); G – giant platelets (%): [17 and 1–90], (mean ± SD: 22.9 ± 20.7); M – Intermediate-sized platelets (%): [44 and 4–72], (mean ± SD: 42.1 ± 14.3); N – normal size platelets (%), [33 and 0–86], (mean ± SD: 35.2 ± 21.1); (*): *P* < 0.0001. (B) Number of patients (*n*) with respect to the platelet count (×10^9^/L) – ≤10: *n* = 8; 11–50: *n* = 48; 51–99: *n* = 40; ≥100: *n* = 11. (C) Percentage of giant platelets (%) with respect to the platelet count (×10^9^/L, *x* axis), [median and range] – ≤10: [32 and 14–90], (mean ± SD: 45 ± 32); 11–50: [23 and 3–82], (mean ± SD: 29 ± 21); 51–99: [13 and 2–56], (mean ± SD: 15 ± 12); ≥100: [6 and 1–27], (mean ± SD: 10 ± 9); (*): *P* = 0.0004. (D) Percentage of intermediate-sized platelets (%) with respect to the platelet count (×10^9^/L) [median and range] – ≤10: [40 and 9–60], (mean ± SD: 38 ± 21); 11–50: [43 and 13–72], (mean ± SD: 42 ± 13); 51–99: [45 and 4–63], (mean ± SD: 43 ± 13); ≥100: [41 and 15–60], (mean ± SD: 38 ± 15); (NS): *P* = 0.88. (E) Percentage of normal-sized platelet (%, *y* axis) with respect to the platelet count (×10^9^/L, *x* axis) [median and range] – ≤10: [18 and 1–36], (mean ± SD: 18 ± 14); 11–50: [26 and 0–70], (mean ± SD: 28 ± 19); 51–99: [37 and 11–86], (mean ± SD: 41 ± 18); ≥100: [58 and 15–82], (mean ± SD: 50 ± 22); (*): *P* = 0.0013.

A “microscope platelet count” was recorded in 48 cases using a calculating chamber as a control of impedance counting, and an “optical platelet count” in 61 cases. The mean platelet count (×10^9^/L) was 53 with a range between under 10 and 136, the highest platelet count being under the normal range (150–400 × 10^9^/L) (Fig. 2A). Four groups of platelet count were defined (Fig. 2B). A majority of patients had a mean platelet count between 11 and 50 × 10^9^/L (44.8%) or between 51 and 99 × 10^9^/L (37.3%). A small number of patients had either a very severe thrombocytopenia (≤10 × 10^9^/L, 7.4%) or, in contrast, a moderate thrombocytopenia (>101 × 10^9^/L, 10.3%).

#### Relationship between the platelet size and the platelet count

Among the 109 patients, 85 were available for the study of platelet size. The percentage (median) of the three groups of platelets defined in Figure 2A was 17, 44, and 33% representing giant, intermediate-sized, and normal-sized platelets, respectively. Among these groups, intermediate-sized platelets were the most abundant (median: 44%; *P* < 0.0001). The percentage of giant, intermediate-sized, and normal-sized platelets was also analyzed according to the platelet count (Fig. 2C–E). Significantly, the lower the platelet count, the higher the percentage of giant platelets (*P* = 0.0004) and the lower the percentage of normal-sized platelets (*P* = 0.0013). In contrast, whatever the platelet count the percentage of intermediate-sized platelets did not vary significantly.

#### Leukocyte myosin inclusions

**Figure 3:**
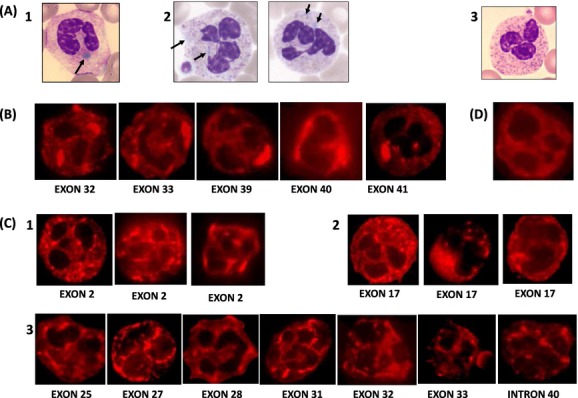
Leukocyte inclusion bodies. (A) MGG blood smears (*n* = 99, positive 92); A1: typical large oval shape inclusions; A2: different aspects of the inclusions; A3: negative control. (B and C) IF blood smears (*n* = 79, positive 73). (B) IF type I; (C) 1–3, IF type II/III: heterogeneous aspects; (D) IF negative control.

Leukocyte myosin inclusions were studied on MGG-stained blood smears in 99 cases (examples in Fig. 3A) and using the IF technique in 79 (examples in Fig. 3B and C). Inclusions were found in 92/99 MGG blood smears. The percentage of positive leukocytes (77/92 available) was: mean 84.6% and range 5–100%. The inclusions were either rare, large, and spindle or round in shape or numerous, small, and punctiform.

Using IF, inclusions were found in 73/79. Inclusions were initially classified according to Kunishima et al. (2003) into three types. In our patients, the identification of type I was easy (Fig. 3B) but the differentiation between types II and III was difficult due to their heterogeneity with regard to the number and size of the spots (Fig. 3C). Therefore, types II and III were merged into type II/III, Among 73 cases, 15 had rare large and spindle/round shape inclusions (type I) and 58 had several or numerous medium/small size, punctiform/speckled inclusions, and were considered as type II/III. Among the six cases where inclusions could not be detected even using IF procedures, one presented abnormal structures using electron microscopy (EM) typical of MYH9-RD.

#### Bleeding

**Figure 4:**
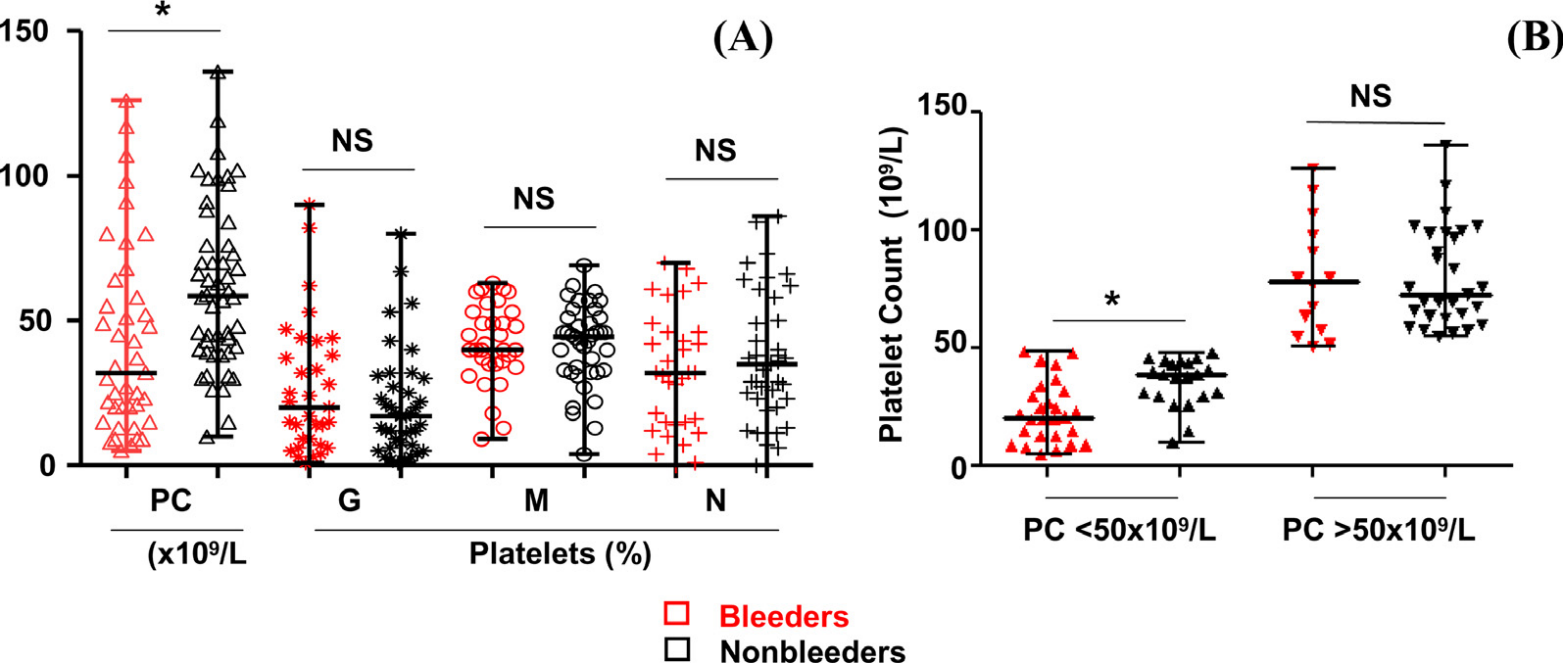
Platelet count (PC × 10^9^/L) and platelet size (G = percentage of giant platelets; M = percentage of intermediate-sized platelets; N = percentage of normal-sized platelets) in bleeders (red symbols) and nonbleeders (black symbols). (A) PC in 41 bleeders versus 50 nonbleeders and platelet size in 33 bleeders versus 40 nonbleeders [median and range] – PC in bleeders [32 and 5–126], (mean ± SD): 42 ± 32,); PC in nonbleeders [58 and 10–136], (mean ± SD: 61 ± 28); (*): *P* = 0.0036. G in bleeders [20 and 1–90], (mean ± SD: 26 ± 22) – G in nonbleeders [17 and 1–80], (mean ± SD: 20 ± 18); (NS): *P* = 0.25. M in bleeders [40 and 9–63], (mean ± SD: 42 ± 13) – M in nonbleeders [44 and 4–69], (mean ± SD: 41 ± 14); (NS): *P* = 0.86. N in bleeders [32 and 0–70], (mean ± SD: 32 ± 20) – N in nonbleeders [35 and 0–86], (mean ± SD: 37 ± 21); (NS): *P* = 0.29. (B) Comparison of PC between bleeders and nonbleeders in two groups defined by a PC under or above 50 × 10^9^/L: under 50 × 10^9^/L:PC in bleeders (*n* = 27), [median and range]: [21 and 5–49], (mean ± SD): 23.1 ± 13) – PC in nonbleeders (*n* = 21), [39 and 10–48], (mean ± SD: 35.76 ± 10.33), *P* = 0.0012; Above 50 × 10^9^/L – PC in bleeders (*n* = 14), [median and range]: [78.5 and 51–126], (mean ± SD): 80.3 ± 24.5); PC in nonbleeders (*n* = 29), [73 and 55–136], (mean ± SD: 80.45 ± 21.10), *P* = 0.825.

In our population, bleeding occurred in 41 patients (45% of 91 available). We compared the platelet count and the platelet size in bleeders and nonbleeders (Fig. 4). The platelet count was significantly lower in bleeders than in nonbleeders, *P* = 0.0036, whereas no significant difference was observed for the platelet size between the two groups (Fig. 4A).

As a platelet count of <50 × 10^9^/L is recommended for measures to prevent bleeding (Slichter 2007), we defined two groups of patients with a platelet count under or above 50 × 10^9^/L. The number of bleeders was significantly higher when the platelet count was under 50 × 10^9^/L (*P* = 0.023). In this group, again the bleeders had a platelet count significantly lower than nonbleeders (*P* = 0.0012). In contrast, no significant difference was observed in platelet count between bleeders and nonbleeders in those patients with a platelet count above 50 × 10^9^/L (*P* = 0.825), (Fig. 4B). Whatever the platelet count, under or above 50 × 10^9^/L, the distribution of the platelet sizes was not significantly different between bleeders and nonbleeders (Fig. S1A and B). These results indicated that bleeding was related to the platelet count but not to the platelet size.

#### Extrahematological symptoms

The number of cases with extrahematological symptoms and age at onset are shown in Figure S2. Among 101 patients available, 41 (40.6%) presented at least one extrahematological symptom. Among these 41 patients, deafness (*n* = 35; 34.70%) was the most frequent, followed by nephropathy (*n* = 21; 20.79%). For those 35 patients with loss of hearing function, 13 needed hearing help. Among the 21 patients with defective kidney function, six had proteinuria, nine had CRF, and six developed ESRF leading to kidney transplantation in the six cases. Cataracts were rare (*n* = 5; 4.9%). The age (years) at onset was significantly lower for deafness (median=16 years; range: 2–60) than for nephropathy (median = 27 years; range: 6–60), *P* = 0.04, (Fig. S2A). Deafness was isolated in 54.28% (*n* = 19) of cases, as compared to nephropathy that was associated with one or two other extrahematological symptoms in a majority of patients (85.71%, *n* = 18) (Fig. S2B).

### Genotype-phenotype correlations

#### Bleeding and *MYH9* affected domain

**Figure 5:**
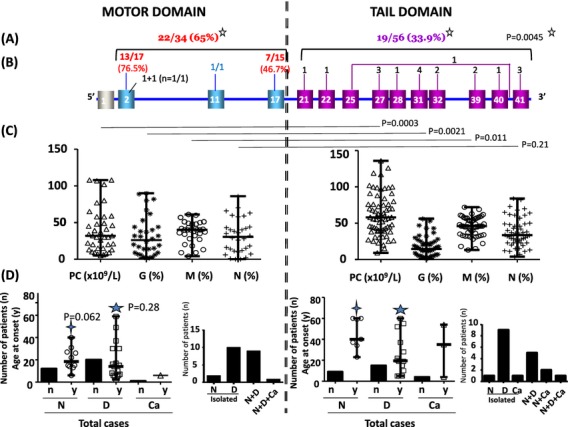
Phenotype given by mutations in the motor and the tail domains. (A) Bleeders according to each domain: number of bleeders/total number of patients available. (B) Number of bleeders for each affected exon and intron. (C) Platelet phenotype: count (PC × 10^9^/L) and size (G = percentage of giant platelets; M = percentage of intermediate-sized platelets; N = percentage of normal-sized platelets) [median and range]: MD – PC [32 and 5–108], (mean ± SD): 39.33 ± 30.15); for 32 out of 39 MD patients, G – [26 and 1–90], (mean ± SD: 31.81 ± 26.96); M [40 and 4–61], (mean ± SD: 36.97 ± 14.70); N – [30.5 and 0–86], (mean ± SD: 31.47 ± 23.33). TD – PC [58 and 9–136], (mean ± SD): 61.07 ± 28.52); for 52 out of 69 TD patients, G – [14.5 and 1–56], (mean ± SD: 17.71 ± 13.54); M [46 and 13–72], (mean ± SD: 45.04 ± 13.39); N [33.5 and 4–84], (mean ± SD: 37.44 ± 19.69). *P* value comparing the platelet phenotype of the two domains is indicated above the scattered dot plot. (D) Extrahematological phenotype: Nephropathy (N), Deafness (D), Cataracts (Ca): Total number of patients (*n*) and age at onset (y = years) [median and range]. MD: Total cases: N (*n* = 12; 31.58%), age (*n* = 10) [18.5 and 6–40] (mean ± SD: 20.9 ± 10.18), D (*n* = 20; 52.63%), age (*n* = 17) [14 and 2–59] (mean ± SD: 18.59 ± 16.74), Ca (*n* = 1; 2.63%), age 6 years. Isolated symptoms: N (*n* = 2; 13.85%), D (*n* = 10; 26.31%). Associated symptoms: N+D (*n* = 9; 23.68%), N+D+Ca (*n* = 1; 2.63%). TD: Total cases: N (*n* = 9; 15.51%), age (*n* = 7) [40 and 23–60] (mean ± SD: 44.71 ± 14.94), D (*n* = 15; 24.19%), age (*n* = 14) [19.5 and 5–60] (mean ± SD: 25.14 ± 18.89), C (*n* = 4; 6.45%), age (*n* = 3) [35 and 4–54] (mean ± SD: 31 ± 25.24). Isolated symptoms: N (*n* = 1; 1.61%), D (*n* = 9; 14.51%), Ca (*n* = 1; 1.61%). Associated symptoms: N+D (*n* = 5; 8.06%), N+Ca (*n* = 2; 3.22%), N+D+Ca (*n* = 1; 1.61%).

We evaluated the number and percentage of bleeders according to the affected domain. MD mutations were associated with a significantly higher percentage of bleeders (65%) as compared to those affecting the TD (33.9%), *P* = 0.045 (Fig. 5A). In MDP, the majority with a mutation in exon 2 and nearly half of the patients with a mutation in exon 17 were bleeders (76.5% and 46.7%, respectively). In TDP a smaller percentage of bleeders was distributed all along the TD (Fig. 5B).

#### Platelet count and size and *MYH9* affected domain

As shown in Figure 5C, we compared the platelet count and size according to the location of the mutation. Patients with mutations in the MD had a significantly lower platelet count (median = 32 × 10^9^/L) and possessed fewer intermediate-sized platelets percentage (median = 40%) than those with a mutation in the TD (median = 58 × 10^9^/L, 46%), *P* = 0.0003 and 0.011, respectively. In contrast, the percentage of giant platelets was significantly higher for MDP than TDP (median = 26% as compared to 14.5%), *P* = 0.0021.

#### IF inclusions and genotype

The aspect of IF inclusions according to the localization of the mutation is presented in Figure 3. Among 15 cases with type I inclusions (Fig. 3B), six had a mutation in exon 39, six in exon 41, one in exons 32, 33, and 40. The 58 cases with type II/III inclusions were distributed among exon 2, 17, 21, 25, 27, 28, 31, 32, 33, and there was a splice site alteration within intron 40 (Fig. 3C1–C3). All mutations affecting exon 39, 40, and 41 were associated exclusively with type I inclusions (Fig. 3B). All mutations affecting the MD and the first exons of the TD until exon 31 were strictly associated with type II/III inclusions. Mutations located in exons 32 and 33 were associated with either type I or type II/III inclusions.

#### Extrahematological symptoms and *MYH9* affected domain

Cases with nephropathy, deafness and cataracts were also studied in relation to the location of the mutation. Each extrahematological symptom was found either alone or associated with one or both of the others. As shown in Figure 5D, 22 from 38 MDP (57.89%) and 19 from 62 TDP (30.64%) presented an additional extrahematological symptom indicating that the frequency of the extrahematological symptoms was higher for MDP, *P* = 0.0115. In addition, the association of nephropathy and deafness were significantly more frequent for MDP than TDP (*P* = 0.042 and 0.0038, respectively). Moreover, nephropathy occurred earlier in MDP (median = 18.5 years) than in TDP (median = 40 years), *P* = 0.062. No significant difference was observed for the age at onset of deafness between MDP and TDP (*P* = 0.28, Fig. 5D).

## Discussion

Since the initial reports of the association of *MYH9* mutations and inherited macrothrombocytopenia, a large number of isolated cases or series have been published. As already mentioned, at least 49 mutations have been identified (Balduini et al. 2013) including those in the most recent publications (Glembotsky et al. 2012; Ishida et al. 2012; De Rocco et al. 2013). This study is the first report of a large cohort of MYH9-RD patients recruited in France since 2002. Our MYH9 cohort included 161 individuals with 37 isolated MYH9-RD cases and 124 subjects from 39 families. Among these 124 subjects, 72 showed macrothrombocytopenia (39 probands and 33 family members) and 52 relatives had a normal platelet count although one suffered from an acquired thrombocytopenia (N. Schlegel and B. Saposnik, unpublished data).

The characteristics of our cohort included the following: (1) homogeneity of the origin (Caucasian for the majority) (2) a high contingent of children (35/109, 32.1%) at enrollment as compared to other series, and (3) the early diagnostic of macrothrombocytopenia (childhood in 52/74 cases: 70.3%). Most frequently, macrothrombocytopenia was detected in systematic screening performed because of infection or prior to surgery, suggesting that the majority of patients did not present a major bleeding syndrome, a finding concordant with the literature for MYH9-RD. Nevertheless, bleeding was the first manifestation of the macrothrombocytopenia in about a third of the cases (22/74; 29.7%) and was observed mainly during childhood (19/22; 86.4% of cases) and before 5 years for a majority (15/19: 78.9%). Bleeding was minor or moderate in most cases. Exceptions seen for five patients included post trauma extradural hematoma at 5 years, amygdalectomy at 4 years, a neonatal parietal hematoma in unknown circumstances, meningeal bleeding at birth, and neonatal bleeding for which no details were available. Neonatal petechial purpura was noted for one case. The platelet count was significantly lower in children with bleeding when compared to those without bleeding, both when investigated in predefined cases or during systematic screening, *P* = 0.035 (data not shown). This tendency was strengthened when comparing the total number of bleeders to the nonbleeders in the whole population, *P* = 0.0043 (data not shown).

Looking at the bleeding events in our cohort after the discovery of the syndrome, the bleeding tendency in 41 bleeders was associated with a lower platelet count (*P* = 0.0036). In order to evaluate the role of platelet size in the bleeding syndrome, the platelet size was compared to the erythrocyte size and three different groups were defined: giant platelets, intermediate-sized platelets, and normal-sized platelets. Our results demonstrate that bleeding was related to the platelet count but not with platelet size, as suggested by several previous reports. It appears then that the presence of giant platelets per se is not a risk factor for bleeding in MYH9-RD.

All patients in our cohort presented a macrothrombocytopenia. A majority of them had a platelet count between 10 and 100 × 10^9^/L. None had a normal platelet count over 150 × 10^9^/L, as has been reported in very rare cases (Pecci et al. 2008). We are aware that hematology analyzers can miss large platelets, underestimating the platelet count. Today, the best way to evaluate low platelet counts, especially when large platelets are present, is still debated. Since the beginning of the study in 2003, we adapted the methods of platelet counting taking into account technical improvements of the analyzers. Initially, results with impedance analyzers were controlled by light microscopic counting. Later, the results from the optical light scatter methods, largely used nowadays, were considered as close as possible to the true platelet count and were recorded as such for our patients. As to the definition of platelet size, we are aware that its comparison to erythrocyte size is an approximation of the platelet diameter only. Nevertheless, using recent bioinformatics procedures and using an image analysis program, we defined three groups: normal, intermediate-sized, and giant platelets corresponding to diameters compatible with results already published (Fig. S3) (Noris et al. 2009). Others have used a software-assisted image analysis system (Noris et al. 2013) but this is only available to a small number of laboratories. In our cohort, intermediate increase in platelet size was the predominant finding (44%) *P* < 0.0001. However, as suggested in the literature (Balduini et al. 2012), our results confirmed that the percentage of giant platelets was significantly higher in the group with a platelet count under 10 × 10^9^/L compared to the three other groups defined in Figure 2 (*P* = 0.0004). This would suggest that megakaryocytes forming giant platelets have insufficient mass to provide normal platelet numbers. We attempted to define a cutoff value of the percentage of giant platelets and intermediate-sized platelets as a help for diagnosis and genetic counseling. In fact, nearly all patients (81/85, 95.3%) had a combined percentage of giant and intermediate-sized platelets that was >30%, irrespective of the proportion of giant platelets. In the four cases (4.7%) with a total percentage of giant and intermediate-sized platelets under 30%, the percentage of giant platelets was always >3%. Therefore, in our cohort, a cutoff value of 30% for both giant plus intermediate-sized platelets or, in rare cases a cutoff value of giant platelets of 3% is always associated with *MYH9* mutations. These cutoff values need to be confirmed in other cohorts and also should be examined in other macrothrombocytopenias such as Bernard-Soulier Syndrome prior to deciding if they constitute a diagnostic step. The mean platelet volume (MPV) was not included in this study for several reasons (Latger-Cannard et al. 2012, 2013). These include (1) MPV was not given by the analyzer in a number of cases, (2) some analyzers do not recognize large/giant platelets and give a false estimation of the MPV, and (3) various analyzers were used by contributors to our study leading consequently to heterogeneity in the results.

Besides macrothrombocytopenia, leukocyte myosin aggregates are also a hallmark of MYH9-RD and the association of these two markers is pathognomonic of this disorder. Unfortunately, it can be difficult to recognize these aggregates on MGG-stained blood smears. For example, in our cohort, they were initially missed for several patients, even by experienced teams. IF is proposed as a reliable and more sensitive technique (Savoia et al. 2010). We obtained concordant positive results between this technique and the genetic analysis for all our patients except for six for whom we could not detect leukocyte inclusions. In five of these cases, the mutations were the following: c.2680G>A (p.E894K), *n* = 1, c.2152C>T (p.R718W) *n* = 2, c.1115A>G (p.Q372R) *n* = 1, and c.2105G>A (p.R702H) *n* = 1. Two mutations, p.E894K and p.Q372R are novel but the two others, p.R718W and p.R702H have been published for several patients and inclusions have been found in all. The difficulty to detect inclusions in cases with R702 mutations such as R702H can be explained by their low polyA(+)RNA content (Kunishima et al. 2007). Nonoptimal preanalytical conditions, unsuccessful permeabilization of the leukocytes might be also at the origin of an inability to visualize the myosin aggregates. Interestingly, in the sixth patient with p.L46F (listed as single nucleotide polymorphism [SNP] in NCBI) abnormal structures typical of MYH9-RD were found using EM (Fig. S4). As described recently, abnormal myosin IIa is responsible for an abnormal distribution of granules and internal membranes in both platelets and megakaryocytes (Chen et al. 2013; Pertuy et al. 2013). One explanation for negative IF results might be that the myosin aggregates were too small to be visualized using the classical IF or cytochemical procedures. Therefore, EM might be helpful in those cases with very faint or undetectable leukocyte inclusions.

The combination of extrahematological symptoms to the hematological anomalies discussed above is the most important clinical picture of MYH9-RD. In our cohort, deafness was the most frequent of these symptoms with an early age of onset (median 16 years) and was isolated in most cases or associated with nephropathy occurring at a later median age of 27 years. These results corroborate those already published (Pecci et al. 2008) with some differences in the percentage and the age at onset of the symptoms.

If we compare the two domains of MYHIIA, there was a more severe clinical picture in general for MD mutations compared to TD mutations: the platelet count was lower, the percentage of giant platelets was higher and the incidence of bleeding was more frequent for MD mutations. Kidney disease associated with deafness was observed in the majority of cases with exon 17 mutations (for p.R702C, p.R702H and p.R718W), and in half of the cases with p.S96L or p.N93K mutations in exon 2. Both patients with p.W33R developed deafness later. As expected, the two patients with p.R705H developed early deafness during childhood as described in DFNA17 (Lalwani et al. 2000), but it is the first time that this mutation has been found associated with macrothrombocytopenia and leukocyte inclusions (Fig. S5). In this patient, direct sequencing has been the only method used. The result has been confirmed on two different blood samplings. The other patients with exon 2 and 17 mutations who have not yet developed extrahematological symptoms are generally young and need a close follow-up. An intermediate phenotype was observed in patients with mutations from exon 21 to 33. Interestingly, no patient with the p.R1165C mutation in exon 27 presented extrahematological symptoms. From exon 39 to 41, the majority of patients did not present extrahematological symptoms except two patients who suffer from deafness, one expressing a splicing defect in intron 40 (c.5765+2T>A) and one with a deletion of one G in exon 41 (p.D1941Mfs*7).

All patients in our cohort, 76 propositi and 33 family members, were heterozygous for a variant in the *MYH9* gene. A total of 43 different genetic variants were found: 40 missense mutations/deletions/insertion and 3 SNPs, p.S1114P, p.L46F and c.5766-3del that will be detailed here below. All the mutations affected positions highly conserved in evolution. A large proportion of the variants reported here (21) are novel. Only 18 mutations identified in our patients have been previously published by others. Taking together the genetic variants in *MYH9* gene that have been already published (Balduini et al. 2013; Jang et al. 2012; Kunishima et al. 2012; Glembotsky et al. 2012; De Rocco et al. 2013; Sun et al. 2013) and those presented in this report, as many as 83 genetic alterations have now been described. The percentage of the genetic variants in our French cohort is 43/83 (51.8%).

None of these genetic variants has been found in our control population (100 subjects) nor are described as SNPs in the NCBI database (http://www.ncbi.nlm.nih.gov/gene/4627) except for p.L46F, c.5766-3del and p.S1114P. The variant p.L46F has been recently reported in NCBI as a SNP with an allelic frequency of 2°/°° or 4°/°° (ESP-cohort-population and CSAgilent, respectively), S1114P has an allelic frequency of 1°/°° (CSAgilent, Massy, France) but no frequency data in the population were reported for c.5766-3del.

Among our three patients with p.L46F, this variant was the only variant in one patient. This patient had a pathological phenotype compatible with MYH9-RD: macrothrombocytopenia and early deafness with onset at 10 years, and abnormal structure typical of MYH9-RD at EM (Fig. S4). The two other patients associated p.L46F and another mutation. One patient associating p.L46F and p.E1841K had a phenotype comparable to those who had inherited p.E1841K alone: moderate thrombocytopenia (platelets: 64 × 10^9^/L), giant platelets (8%), and intermediate-sized platelets (54%), type I leukocyte inclusions, but no deafness, nephropathy or cataracts. Therefore, it appeared that the association of p.L46F with p.E1841K did not aggravate the clinical symptoms associated with p.E1841K alone. The other patient with the double alteration p.S96L and p.L46F had a phenotype compatible with p.S96L alone, with major thrombocytopenia (platelets <10 × 10^9^/L), a majority of giant platelets (90%), very pale and small leukocyte inclusions (not detected initially), frequent bleeding episodes, early hearing loss onset at 2 years and nephropathy onset at 40 years.

We confirmed that the c.5766-3del had no effects on *MYH9* transcription (data not shown) and should be considered as a SNP.

Four patients of the same family were heterozygous for the SNP c.3340T>C (p.S1114P) but also a true mutation c.4270G>A (p.D1424N) localized in two different exons (26 and 31, respectively), and did not present a phenotype more severe than patients with c.4270G>A (p.D1424N) mutation alone. Until now only two cases, each with two mutations, have been reported. One patient, a 27-year-old woman, had c.2728A>C in exon 22 (p.K910Q) and c.4270G>C in exon 31 (p.D1424N), probably present in different chromosomes (Capria et al. 2004). She presented a typical phenotype of Fechtner syndrome, as observed in patients with mutations in exon 22 and/or 31. The other patient, a 5-year-old girl, had c.99G>T (p.W33C) and c.103C>G (p.P35A), both in exon 2 (Miyajima and Kunishima 2009). Here the two mutations were located on the same chromosome. The leukocyte inclusions of this patient were of type II and it was suggested that the double mutation might have increased myosin aggregate formation.

A deletion of 21 nucleotides, c.3202_3222del in exon 24 (p.Q1068_L1074 del), was identified in a 59 year-old-woman who had a moderate thrombocytopenia (platelet count: 50–90 × 10^9^/L) but spontaneous and provoked bleeding, large platelets (giant: 46%, intermediate-size increase: 47%), leukocyte inclusions type II/III, hearing loss since childhood, congenital cataracts (operated at 4 years), and mild proteinuria (0.22 g/L). Her son (not included in the present report) had a similar phenotype but no bleeding tendency and no cataracts. The p.Q1068_L1074del was associated with c.5766-3del in the mother but p.Q1068_L1074del was the only genetic alteration in her son. Interestingly, the phenotype of the mother is more severe than that of her son. The p.Q1068_L1074del has also been reported recently in a young Japanese woman, 27 year-old, who already has end-stage renal disease, bilateral hearing loss, but no cataracts (Ishida et al. 2012). Her thrombocytopenia (55 × 10^9^/L) was initially similar to that of our patient, with large platelets and type I leukocyte inclusions. But the kidney impairment was more severe with an earlier onset and a severe evolution, and a recent platelet count decrease to 29 × 10^9^/L. The bleeding tendency and cataracts were absent. Therefore, beside similarities in the phenotype of the three patients, differences in bleeding diathesis and kidney alteration were present. The heptad repeat pattern of the residues plays a crucial role in the function of NMMHC-IIA (Franke et al. 2005). Consequently, the deletion of such a heptad may alter the formation of myosin bipolar filaments. However, the genetic mechanism leading to the heptad deletion remains to be determined. At present, a total of 10 patients, including our two patients, have a deletion or duplication in exon 25 that is considered a hot spot (Ishida et al. 2012). Cases are sporadic (*n* = 5) or familial (*n* = 5).

**Figure 6:**
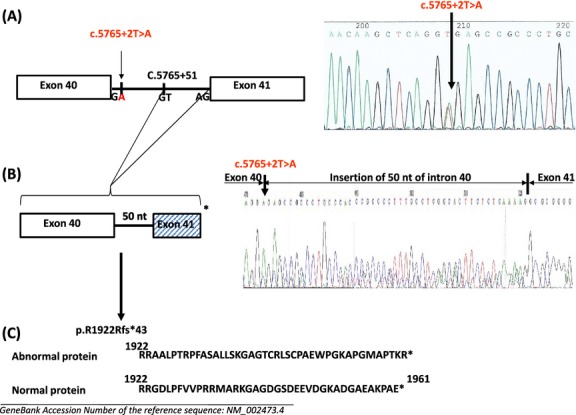
Genetic and molecular mechanism of the missense mutation c.5765+2T>A at the donor splice site of intron 40. (A) DNA sequence analysis. The exons 40 and 41 and the exons-intron boundaries were PCR amplified from genomic DNA and amplified products were directly sequenced, showing a c.5765+2T>A substitution at the donor splice site GT of intron 40. The arrow shows the location of the substitution and, as a consequence, the exposure of a cryptic GT site at position c.5765+51. (B) cDNA analysis. cDNA was obtained from leukocytes and platelets and the sequences corresponding to exons 40–41 were analyzed. The T to A substitution at the splice donor site of intron 40 led to an insertion in mRNA of 50 nts from intron 40, a frameshift of exon 41 and a stop codon TAG after 124 nt. Same results were obtained from leukocytes and platelets cDNA. (C) Predicted protein: The predicted abnormal sequence p.R1922Rfs*43 is shown as compared to the wild-type one.

Finally, for the first time, a variant c.5765+2T>A, a new missense mutation, was localized in the donor splice site GT of intron 40 and was investigated by studying leukocyte and platelet mRNA after reverse transcriptase-PCR amplification followed by sequencing of the amplified fragments. This substitution T>A induces the exposition of a cryptic splice donor site GT at the position c.5765+51 leading to an insertion in mRNA of 50 nucleotides from intron 40 and a premature stop codon in exon 41. The missense mutation induces a frameshift and predicts an abnormal protein p.R1922Rfs*43 (Fig. 6).

In conclusion, our cohort greatly extends the number of mutations known for MYH9-RD. Our group shows considerable clinical heterogeneity with predominantly moderate bleeding, leukocyte inclusion bodies in the majority of cases and the frequent development of extrahematological symptoms with age. We also found a majority of missense mutations, with six exons preferentially affected (2, 17, 27, 31, 39, 41), rare small deletions in exon 25 and nonsense and frameshift alterations restricted to exon 41. The phenotype-genotype correlation showed a higher severity for MD mutations than TD mutations and an intermediate phenotype for mutations from exon 21 to 33. Interestingly some patients with MD mutations did not present extrahematological symptoms that were also seen in a few patients with TD alterations, suggesting that not only age but also other unknown factors might influence MYH9 phenotype.

## Appendix: The French MYH9 Network: list of the participants

*Marie-Christine Alessi***,** Laboratoire d'Hématologie CHU La Timone Marseille; *Magda Alexis*, Service d'Oncologie-Hématologie CHR d'Orléans; *Sylvia Bellucci*, Laboratoire d'Hématologie CHU Lariboisière Paris; *Philippe Beurrier*, Centre Traitement Hémophilie CHU d'Angers; *Catherine Boinot*, Laboratoire d'Hématologie CHU La Milétrie Poitiers; *Jeanne-Yvonne Borg*, Unité Hémostase Hématologie CHU de Rouen; *Alain Bourguignat*, Service d'Onco-Hématologie CHI Meulan Les Mureaux; *Hilaire Charlanne*, Service de Médecine Interne Hôpital Claude Huriez CHRU de Lille; *Karine Dahan*, Service de Néphrologie CHU Henri Mondor Créteil; *Emmanuel De Maistre*, CRTH et Centre des Coagulopathies CHU de Dijon; *Anne Deville*, Service d'Hémato Cancérologie Pédiatrie CHU de Nice; *Marie Dreyfus*, Laboratoire d'Hématologie CHU Le Kremlin Bicêtre; *Carole Fagnou*, Service de Pédiatrie CHU Ambroise Paré Boulogne Billancourt; *Rémi Favier*, Laboratoire d'Hématologie CHU Armand Trousseau Paris; *Maxence Ficheux*, Service de Néphrologie CHU de Caen; *Béatrice Fimbel*, Laboratoire d'Hémostase Hôpital Trousseau CHRU de Tours; *Claire Flaujac*, Service d'Hématologie Biologique CHU Cochin Paris; *Dominique Fleury*, Service de Néphrologie CH Valenciennes; *Alain-Charles Fouilhoux*, Service de Médecine Polyvalente CH Guy Thomas Riom; *Nathalie Hézard*, Laboratoire d'Hématologie CHU Robert Debré Reims; *Marie-Hélène Horellou*, Service d'Hématologie Biologique CHU Cochin Paris; *Alain Kitzis*, Laboratoire de génétique cellulaire et moléculaire CHU de Poitiers; *Wladimir Kohn*, Service de Néphrolo-gie-Hémodialyse Clinique de Tournan; *Véronique Latger-Cannard*, CHU Srvice d'Hématologie Biologique CHU Nancy; *Thierry Leblanc*, Service d'Hématologie Clinique CHU Robert Debré Paris; *Guy Leverger*, Service d'Hématologie et Oncologie Pédiatrique CHU Armand Trousseau Paris; *Vincent Leymarie*, Laboratoires d'Analyses Médicales Brive; *Chantal Loirat*, Service de Néphrologie Pédiatrique CHU Robert Debré Paris; *Dominique Martin-Coignard*, Laboratoire de Génétique CH Le Mans; *Sandra Mercier*, Service de Génétique Médicale Hôpital Sud Rennes; *Gilles Morin*, Génétique Clinique Département de Pédiatrie CHU d'Amiens; *Philippe N'Guyen*, Laboratoire d'Hématologie CHU Robert Debré Reims; *Charles Nasr*, Unité de Néonatologie CH de Sens; *Brigitte Pan-Petesch*, CHU Hôpital Morvan CHU Brest; *Isabelle Pellier*, Service d'Hémato Onco Immuno pédiatrique CHU d'Angers; *Corinne Pondarré*, Service d'immuno-hématologie pédiatrique et de transplantation de moelle osseuse Institut d'Hématologie et d'Oncologie pédiatrique CHU Lyon Est; *Claire Pouplard*, Service Hémato-Hémostase Centre Traitement Hémophilie Hôpital Trousseau CHRU Tours; *Claire Pouteil-Noble*, Service de Néphrologie CHU Lyon Sud; *Catherine Pouymayou*, Laboratoire d'Hématologie CHU La Timone Marseille; *Valérie Roussel-Robert*, Centre des Hémophiles CHU Cochin Paris; *Bruno Royer*, Service d'Hématologie Clinique CHU Amiens; *Lucia Rugeri*, Laboratoire d'Hémostase CHU Lyon; *Yoland Schoindre*, Service de Néphrologie CHU Necker-Enfants Malades Paris; *Pierre Sié*, Laboratoire d'Hématologie CHU Purpan Toulouse; *Alexia Thannberger*, Laboratoire d’ Hématologie CH de la Côte Basque Bayonne; *Catherine Trichet*, Service de Biologie Clinique CH Victor Dupouy Argenteuil; *Nathalie Trillot*, Laboratoire d'Hématologie Hôpital Cardiologique CHRU Lille; *Erwan Choblet*, Laboratoire de Biochimie et d'Hémostase, CHT Gaston Bourret Nouméa Nouvelle Calédonie; *Julie Désir*, Service de Génétique Hôpital Erasme Bruxelles Belgique; *Alina Ferster* and *Yves Sznajer*, Unité de Génétique Clinique Hôpital Universitaire des Enfants Reine Fabiola Bruxelles Belgique.

## Supporting Information

**Figure S1.** Platelet size (G, giant platelets; M, intermediate-sized platelets; N, normal-sized platelets) in bleeders (red symbols) and nonbleeders (black symbols) according to a platelet count (PC) under or over 50 × 10^9^/L. (A) Platelet size in 22 bleeders versus 16 nonbleeders in patients with PC under 50 × 10^9^/L: G in bleeders [32 and 3–90], (mean ± SD: 33 ± 23, data not shown); G in nonbleeders [22 and 5–80], (mean ± SD: 29 ± 22, data not shown); (NS): *P* = 0.48. M in bleeders [40 and 9–61], (mean ± SD: 39 ± 14); M in nonbleeders [45 and 20–69], (mean ± SD: 45 ± 12); (NS): *P* = 0.22. N in bleeders [23 and 0–70], (mean ± SD: 27 ± 20); N in nonbleeders [24 and 0–64], (mean ± SD: 24 ± 16); (NS): *P* = 0.87. (B) Platelet size in 11 bleeders versus 24 nonbleeders in patients with PC over 50 × 10^9^/L: G in bleeders [9 and 1–25], (mean ± SD: 11 ± 7); G in nonbleeders [11 and 1–56], (mean ± SD: 15 ± 13); (NS): *P* = 0.63. M in bleeders [45 and 31–63], (mean ± SD: 47 ± 17); M in nonbleeders [42 and 4–62], (mean ± SD: 39 ± 14); (NS): *P* = 0.18. N in bleeders [43 and 15–68], (mean ± SD: 42 ± 16); N in nonbleeders [39 and 11–86], (mean ± SD: 45 ± 21); (NS): *P* = 0.74.

**Figure S2.** Extrahematological symptoms in 101 available patients. (A) Number of patients (*n*) and age at onset (y = years) [median and range]: Nephropathy (N) (*n* = 21), [27 and 6–60] (mean ± SD: 30 ± 16), Deafness (D) (*n* = 35) [16 and 2–60] (mean ± SD: 21 ± 17), Cataracts (Ca) (*n* = 5) [20 and 4–54] (mean ± SD: 24 ± 24). (B) Number of patients (total = 41) with N, D or Ca isolated, or an association of two or three extrahematological symptoms N alone = 3, D alone = 19, Ca alone = 1, N+D = 14, N+Ca = 2, N+D+Ca = 2.

**Figure S3.** Platelet diameter using the Calopix image analysis program. Comparison of the diameter (*μ*m) of what we have defined as normal, intermediate-sized, and giant platelets to the mean erythrocyte diameter. Normal erythrocyte diameter (mean): between 6 and 7. Diameter of “normal platelets”: <3.50; example: 1.59. Diameter of “intermediate-sized platelets”, defined by a size comprised between half erythrocyte and erythrocyte (between 3.5 and 7); examples: 3.75, 5.22, 5.87. Diameter of giant platelets, defined by a size equal or over the erythrocyte one (≥7); examples: 7.24, 8.36.

**Figure S4.** Electron microscopy of platelets from a patient with the L46F variant showing enlarged round platelets with abnormal distribution of alpha granules present in the periphery of the platelets and the membrane complexes in the center. MC, membrane complex; G, granules. Patient: macrothrombocytopenia, platelet count: 90 × 10^9^/L, hearing loss onset at 10 years, menometrorrhagias.

**Figure S5.** Two patients with p.R705H mutation: giant platelets and leukocyte inclusions. Patient 1: macrothrombocytopenia, platelet count: 107 × 10^9^/L, deafness onset: 7 years and hearing help, epistaxis; patient 2: macrothrombocytopenia, platelet count: 67–100 × 10^9^/L, deafness onset: 14 years, epistaxis; arrows show the leukocyte inclusions.

**Table S1.** Age and circumstances of discovery of macrothrombocytopenia (MT) in 74 available cases: (A) Age (y = years) at MT discovery. (B) Number of patients according to circumstances of MT discovery: B, bleeding; FS, familial study; SS, systematic study.
