# Supplementary figures and images for: Mutation spectrum and genotype-phenotype correlations in a large French cohort of MYH9-Related Disorders
Source: Mol Genet Genomic Med. 2014 Feb 7;2(4):297–312. doi: 10.1002/mgg3.68 (PMC4113270; doi:10.1002/mgg3.68)

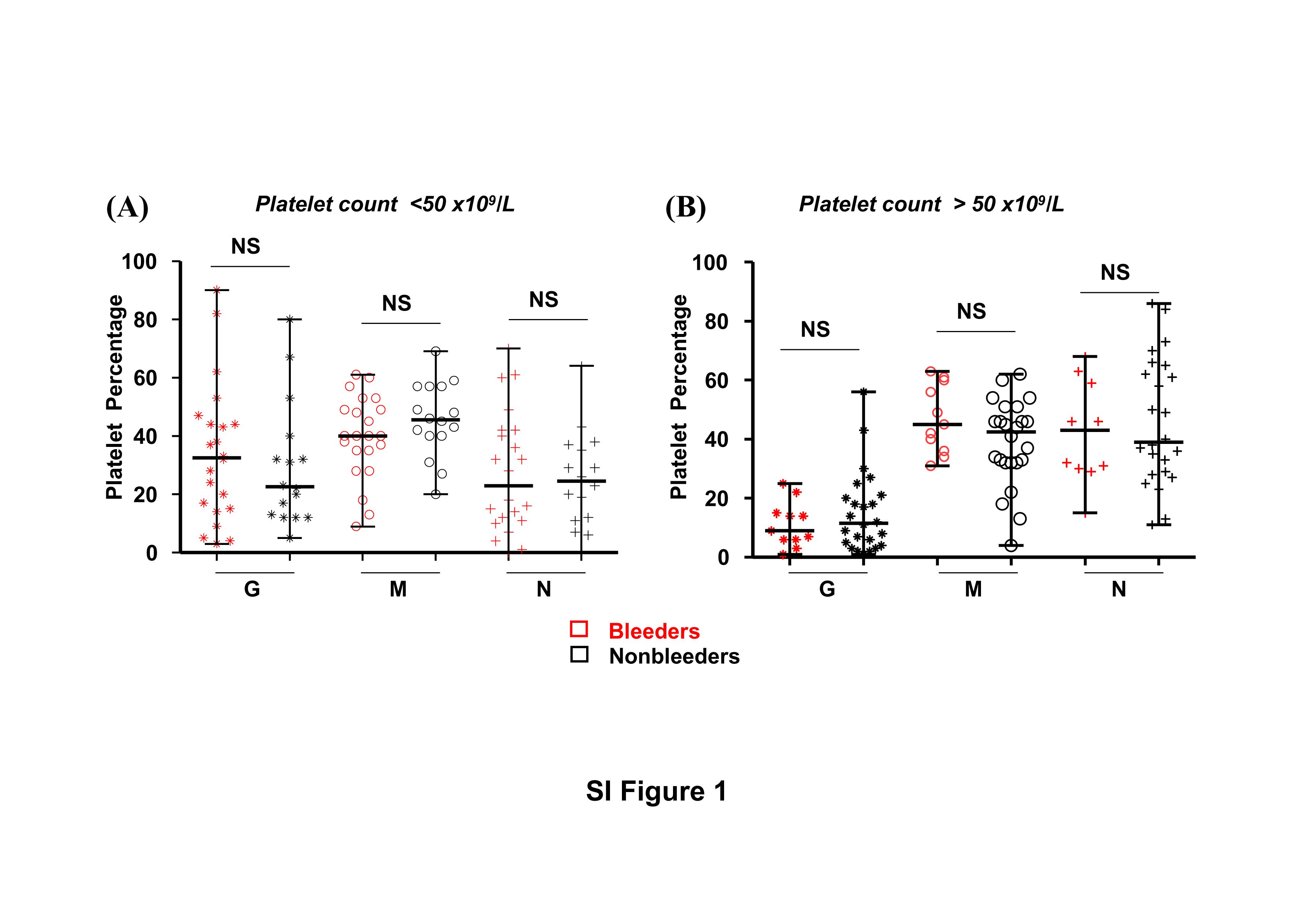

Supplement: Supplementary file 1 — Figure S1. Platelet size (G, giant platelets; M, intermediate-sized platelets; N, normal-sized platelets) in bleeders (red symbols) and nonbleeders (black symbols) according to a platelet count (PC) under or over 50 × 109/L. (A) Platelet size in 22 bleeders versus 16 nonbleeders in patients with PC under 50 × 109/L: G in bleeders [32 and 3–90], (mean ± SD: 33 ± 23, data not shown); G in nonbleeders [22 and 5–80], (mean ± SD: 29 ± 22, data not shown); (NS): P = 0.48. M in bleeders [40 and 9–61], (mean ± SD: 39 ± 14); M in nonbleeders [45 and 20–69], (mean ± SD: 45 ± 12); (NS): P = 0.22. N in bleeders [23 and 0–70], (mean ± SD: 27 ± 20); N in nonbleeders [24 and 0–64], (mean ± SD: 24 ± 16); (NS): P = 0.87. (B) Platelet size in 11 bleeders versus 24 nonbleeders in patients with PC over 50 × 109/L: G in bleeders [9 and 1–25], (mean ± SD: 11 ± 7); G in nonbleeders [11 and 1–56], (mean ± SD: 15 ± 13); (NS): P = 0.63. M in bleeders [45 and 31–63], (mean ± SD: 47 ± 17); M in nonbleeders [42 and 4–62], (mean ± SD: 39 ± 14); (NS): P = 0.18. N in bleeders [43 and 15–68], (mean ± SD: 42 ± 16); N in nonbleeders [39 and 11–86], (mean ± SD: 45 ± 21); (NS): P = 0.74. [file mgg30002-0297-SD1.tif]

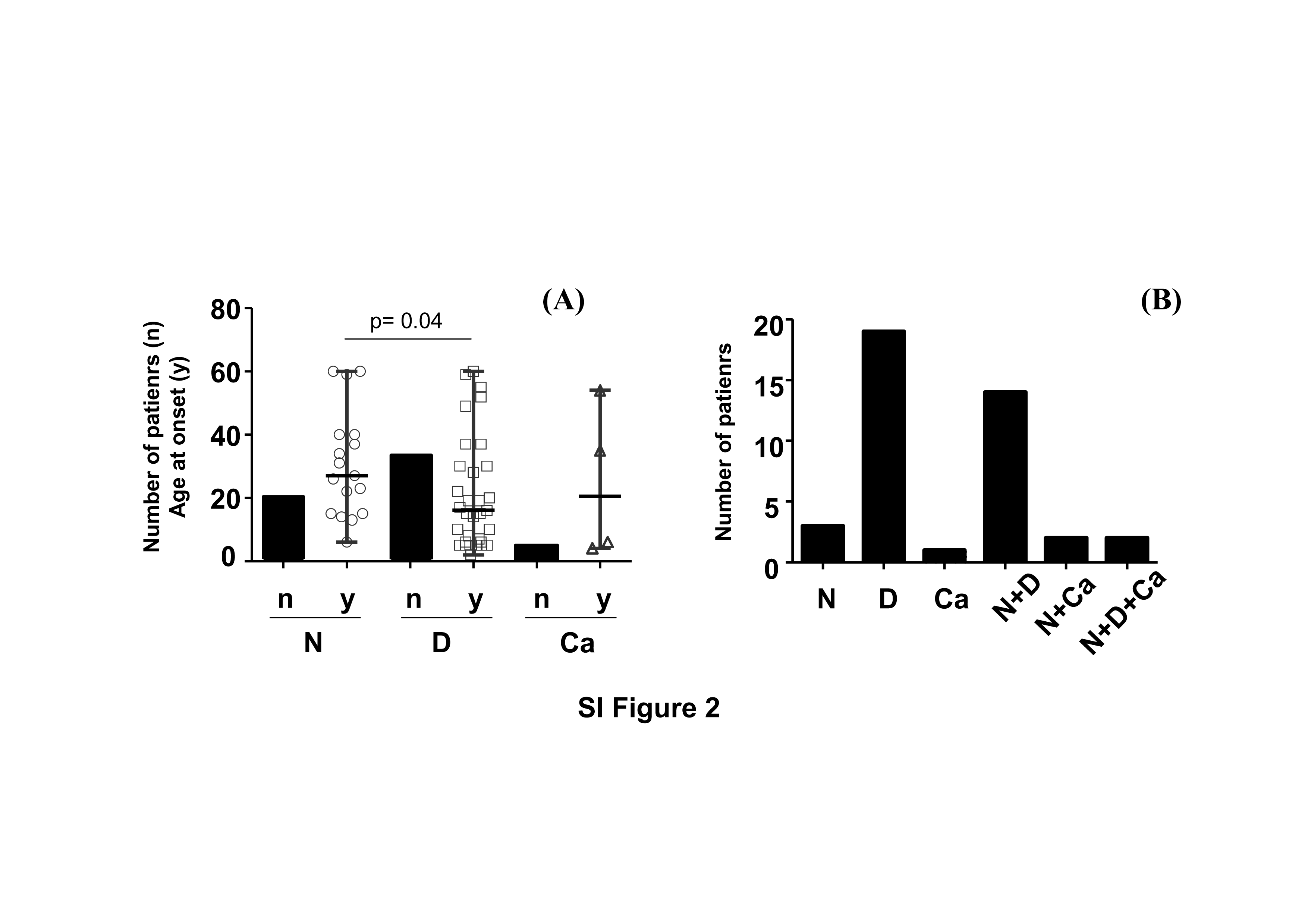

Supplement: Supplementary file 2 — Figure S2. Extrahematological symptoms in 101 available patients. (A) Number of patients (n) and age at onset (y = years) [median and range]: Nephropathy (N) (n = 21), [27 and 6–60] (mean ± SD: 30 ± 16), Deafness (D) (n = 35) [16 and 2–60] (mean ± SD: 21 ± 17), Cataracts (Ca) (n = 5) [20 and 4–54] (mean ± SD: 24 ± 24). (B) Number of patients (total = 41) with N, D or Ca isolated, or an association of two or three extrahematological symptoms N alone = 3, D alone = 19, Ca alone = 1, N+D = 14, N+Ca = 2, N+D+Ca = 2. [file mgg30002-0297-SD2.tif]

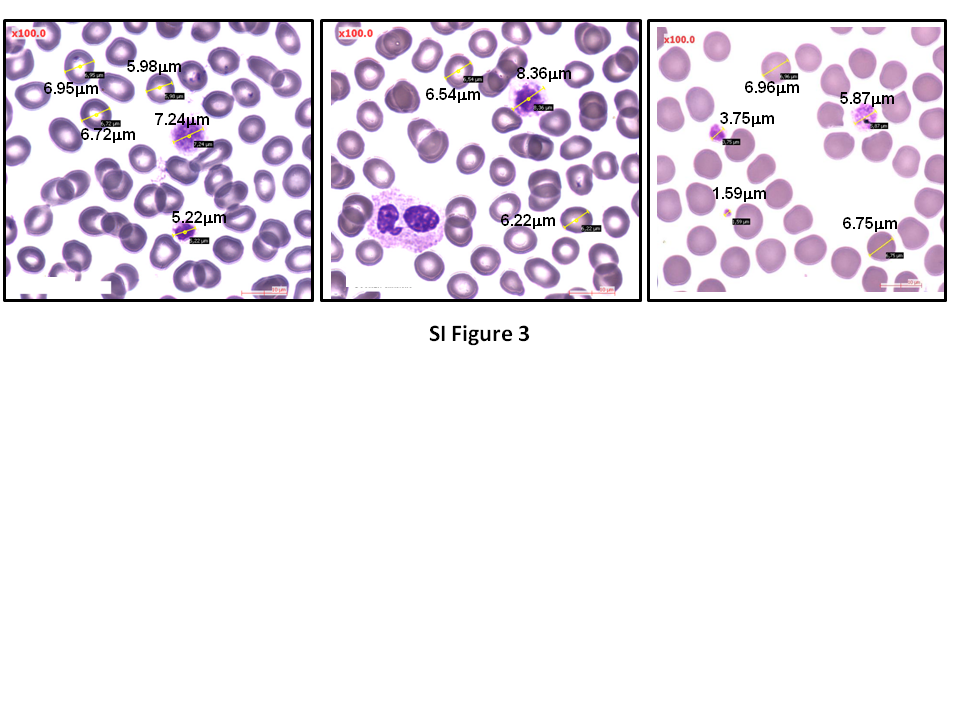

Supplement: Supplementary file 3 — Figure S3. Platelet diameter using the Calopix image analysis program. Comparison of the diameter (μm) of what we have defined as normal, intermediate-sized, and giant platelets to the mean erythrocyte diameter. Normal erythrocyte diameter (mean): between 6 and 7. Diameter of “normal platelets”: <3.50; example: 1.59. Diameter of “intermediate-sized platelets”, defined by a size comprised between half erythrocyte and erythrocyte (between 3.5 and 7); examples: 3.75, 5.22, 5.87. Diameter of giant platelets, defined by a size equal or over the erythrocyte one (≥7); examples: 7.24, 8.36. [file mgg30002-0297-SD3.tif]

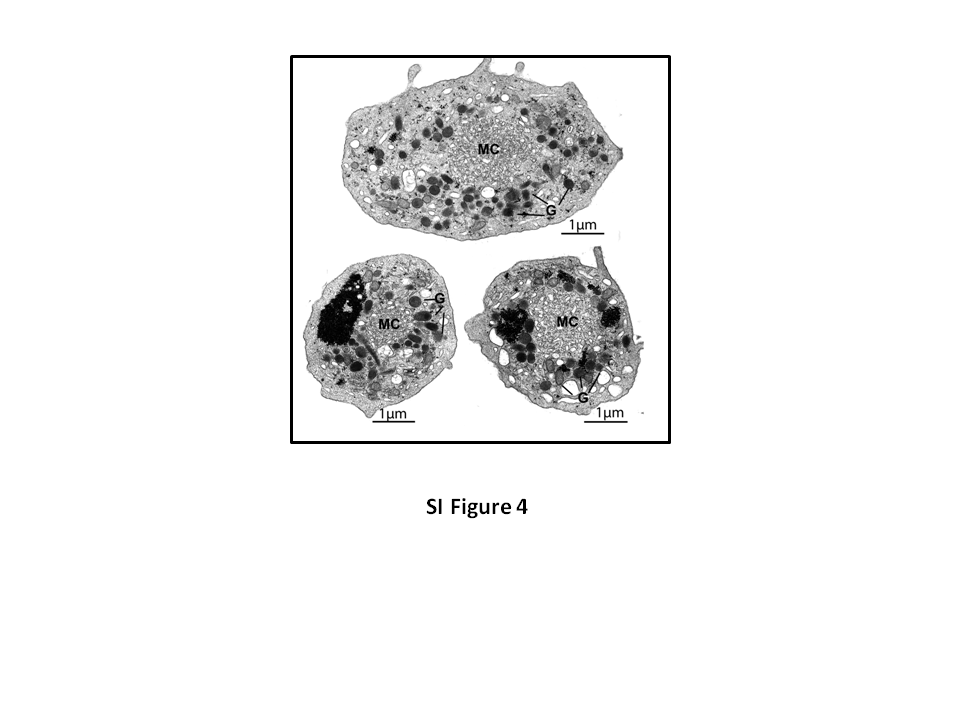

Supplement: Supplementary file 4 — Figure S4. Electron microscopy of platelets from a patient with the L46F variant showing enlarged round platelets with abnormal distribution of alpha granules present in the periphery of the platelets and the membrane complexes in the center. MC, membrane complex; G, granules. Patient: macrothrombocytopenia, platelet count: 90 × 109/L, hearing loss onset at 10 years, menometrorrhagias. [file mgg30002-0297-SD4.tif]

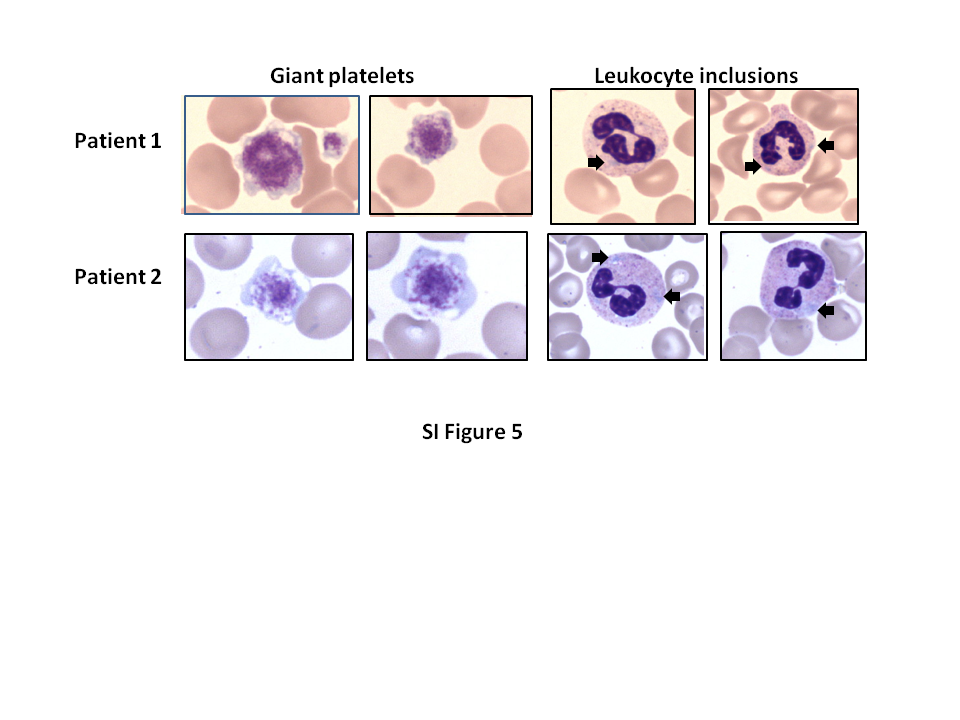

Supplement: Supplementary file 5 — Figure S5. Two patients with p.R705H mutation: giant platelets and leukocyte inclusions. Patient 1: macrothrombocytopenia, platelet count: 107 × 109/L, deafness onset: 7 years and hearing help, epistaxis; patient 2: macrothrombocytopenia, platelet count: 67–100 × 109/L, deafness onset: 14 years, epistaxis; arrows show the leukocyte inclusions. [file mgg30002-0297-SD5.tif]
