# Supplementary material for: Mutation spectrum and genotype-phenotype correlations in a large French cohort of MYH9-Related Disorders
Source: Mol Genet Genomic Med. 2014 Feb 7;2(4):297–312. doi: 10.1002/mgg3.68 (PMC4113270; doi:10.1002/mgg3.68)
Supplement: Supplementary file 6 — Table S1. Age and circumstances of discovery of macrothrombocytopenia (MT) in 74 available cases: (A) Age (y = years) at MT discovery. (B) Number of patients according to circumstances of MT discovery: B, bleeding; FS, familial study; SS, systematic study. [file mgg30002-0297-SD6.doc]

**SI Table I**

**A**

| **Age at MT discovery** | **Total** | **Propositi** | **Family members** |
| --- | --- | --- | --- |
| **0-5 y** | **39** | **27** | **12** |
| **6-18 y** | **13** | **9** | **4** |
| **Adults** | **22** | **13** | **9** |
| **All ages** | **74** | **49** | **25** |

**B**

| **Patients**  **MYH9-RD** | **B** | **FS** | **SS** |
| --- | --- | --- | --- |
| **0-5 y** | **15** | **8** | **16** |
| **6-18 y** | **4** | **2** | **7** |
| **Adults** | **3** | **8** | **11** |
| **All ages** | **22** | **18** | **34** |
